# The Two Faces of Tumor-Associated Macrophages and Their Clinical Significance in Colorectal Cancer

**DOI:** 10.3389/fimmu.2019.01875

**Published:** 2019-08-20

**Authors:** Marta L. Pinto, Elisabete Rios, Cecília Durães, Ricardo Ribeiro, José C. Machado, Alberto Mantovani, Mário A. Barbosa, Fatima Carneiro, Maria J. Oliveira

**Affiliations:** ^1^i3S-Instituto de Investigação e Inovação em Saúde, Universidade do Porto, Porto, Portugal; ^2^INEB-Institute of Biomedical Engineering, University of Porto, Porto, Portugal; ^3^Institute of Biomedical Sciences Abel Salazar (ICBAS), University of Porto, Porto, Portugal; ^4^CNC-Center for Neuroscience and Cell Biology, University of Coimbra, Coimbra, Portugal; ^5^IPATIMUP-Institute of Molecular Pathology and Immunology of the University of Porto, Porto, Portugal; ^6^Department of Pathology, Faculty of Medicine, University of Porto, Porto, Portugal; ^7^Department of Pathology, Centro Hospitalar São João, Porto, Portugal; ^8^Laboratory of Genetics and Environmental Health Institute, Faculty of Medicine, University of Lisbon, Lisbon, Portugal; ^9^Department of Clinical Pathology, Centro Hospitalar e Universitário de Coimbra, Coimbra, Portugal; ^10^Humanitas Clinical and Research Center, Milan, Italy; ^11^Humanitas University, Milan, Italy

**Keywords:** colorectal cancer, tumor immunomodulation, tumor-associated macrophages, human macrophage surface markers, macrophage polarization, prognostic and tumor relapse

## Abstract

Macrophages are one of the immune populations frequently found in colorectal tumors and high macrophage infiltration has been associated with both better and worst prognosis. Importantly, according to microenvironment stimuli, macrophages may adopt different polarization profiles, specifically the pro-inflammatory or M1 and the anti-inflammatory or M2, which display distinct functions. Therefore, concomitantly with the number of tumor-associated macrophages (TAMs), their characterization is fundamental to unravel their relevance in cancer. Here, we profiled macrophages in a series of 150 colorectal cancer (CRC) cases by immunohistochemistry, using CD68 as a macrophage lineage marker, CD80 as a marker of pro-inflammatory macrophages, and CD163 as a marker of anti-inflammatory macrophages. Quantifications were performed by computer-assisted analysis in the intratumoral region, tumor invasive front, and matched tumor adjacent normal mucosa (ANM). Macrophages, specifically the CD163^+^ ones, were predominantly found at the tumor invasive front, whereas CD80^+^ macrophages were almost exclusively located in the ANM, which suggests a predominant anti-inflammatory polarization of TAMs. Stratification according to tumor stage revealed that macrophages, specifically the CD163^+^ ones, are more prevalent in stage II tumors, whereas CD80^+^ macrophages are predominant in less invasive T1 tumors. Specifically in stage III tumors, higher CD68, and lower CD80/CD163 ratio associated with decreased overall survival. Importantly, despite the low infiltration of CD80^+^ cells in colorectal tumors, multivariate logistic regression revealed a protective role of these cells regarding the risk for relapse. Overall, this work supports the involvement of distinct microenvironments, present at the intra-tumor, invasive front and ANM regions, on macrophage modulation, and uncovers their prognostic value, further supporting the relevance of including macrophage profiling in clinical settings.

## Introduction

A variety of non-malignant stromal cells present at the complex tumor microenvironment are active players in cancer progression (1). Specifically in solid tumors, tumor associated macrophages (TAMs) are one of the most represented populations (2) and have important roles in the invasive, angiogenic, and metastatic processes (3, 4).

Macrophages are extremely plastic cells that are able to respond and adapt to external stimuli (5). Currently, the most accepted model of macrophage classification describes several polarization statuses between two extreme populations: the M1-like or pro-inflammatory, and the M2-like or anti-inflammatory. In the presence of factors such as lipopolysaccharide (LPS), interferon (IFN)-γ or tumor necrosis factor (TNF)-α (6), macrophages adopt a pro-inflammatory phenotype, with high antigen presenting capacity and production of cytokines such as interleukin (IL)-6, IL-12, TNF-α, IFN-γ, and reactive oxygen species (ROS). These cells are known for their bactericidal and pro-inflammatory functions (7). On the other extreme of the spectrum are the M2-macrophages, induced by factors such as IL-4, IL-13, IL-10 or glucocorticoids, which produce anti-inflammatory cytokines, specifically transforming growth factor (TGF)-β and IL-10 (8). They are characterized by their scavenger, angiogenic, and pro-invasive properties (3, 4). As a consequence of the immunosuppressive tumor microenvironment, namely due to high IL-10 and TGF-β levels (9, 10), TAMs are reported to adopt features common to M2-like macrophages. They generally produce growth factors, chemokines, and matrix metalloproteinases (MMPs), which act directly on cancer cells or in other stromal cells, ultimately leading to tumor growth, invasion, and metastasis (3).

Several clinical and epidemiological studies have described a strong association between TAMs infiltration, worst prognosis and shorter survival in melanoma, breast, and ovarian cancer (11–15). In the specific case of colorectal cancer (CRC), some studies conclude that higher macrophage infiltration correlates with more advanced tumor stages (16) and worst prognosis (17), while others report that TAMs are associated with improved survival, specifically in the colon (18), and with reduced liver metastasis (19). Taken together, these findings suggest lack of agreement on the role of TAMs on CRC clinical course. Importantly, the majority of these studies were solely based on CD68, a macrophage lineage marker, without taking into consideration differences amongst the distinct pro- or anti- inflammatory subpopulations. Recognizing the importance of macrophage polarization, some authors analyzed markers which discriminate between M1 and M2 subpopulations. In this sense, Algars et al. (20) recently proposed that the type and distribution of TAMs may influence the carcinogenic process, ultimately affecting survival. In less advanced tumor stages, macrophage infiltration was associated with improved disease free survival, whereas, in stage IV CRC, high number of CLEVER-1/Stabilin-11^+^ cells, used as an M2-marker, correlated with shorter disease-free survival (20). A recent meta-analysis performed in head and neck squamous cell carcinoma reinforced the need to evaluate macrophage subsets: CD68 did not present any prognostic association, contrarily to what was observed for CD163 which correlated with decreased survival (21). Nevertheless, in both studies, anti-inflammatory macrophages were not evaluated. Reports using nitric oxide synthase 2 (NOS2) as a M1 macrophages marker and CD163 as a M2 macrophage marker, yielded controversial results (22, 23). Although NOS2 has been frequently used to identify pro-inflammatory macrophages in mice, many research groups argued that differences in human nitric oxide metabolism likely preclude using it as an appropriate marker to identify M1 macrophages (24–26). Other limitations of published studies are related to the use of tissue microarrays (which may not accurately represent the characteristics of the tumor), the evaluation of hotspots (an approach that already presents some bias in the analysis) and the use of a semiquantitative scoring (which results in more subjective and less sensitive method).

In this study we performed a quantitative evaluation of the distinct macrophage subpopulations present in CRC, using CD68, CD80 and CD163 lineage, pro- and anti-inflammatory surface markers, respectively, in consecutive histological slides. Quantifications were performed in the intratumoral region (IT), tumor invasive front (IF), and tumor adjacent normal mucosa (ANM) of the same patient, to elucidate how the distinct region microenvironments may modulate macrophages. Histological profiling was then combined with clinicopathological and follow-up data, in order to unravel the clinical impact of distinct macrophage subpopulations within colorectal tumors, and discriminate which patients may benefit from immunotherapies targeting macrophages.

## Materials and Methods

### Clinical Samples

One hundred and fifty CRC primary tumors (83 males and 67 females, median age 70.5 years old, range 22–93 years), containing in the same histological section tumor and normal mucosa, were retrieved from the files of the Pathology Department from Centro Hospitalar Universitário São João (CHUSJ, Porto, Portugal). Samples were collected during primary tumor surgical resections between 2007 and 2012. Synchronous tumors were not included.

All clinicopathological evaluations, including stage, grade, tumor type and lymphocytic infiltrate, were performed by experienced pathologists from the CHUSJ Pathology Department and are included in Table 1. The existence of tumor relapses, the therapeutic scheme and patient overall survival is also included. In this retrospective cohort, only five patients received pre-operative chemotherapy, of which three also received pre-operative radiotherapy. From the initial cohort, clinical data for survival analyses was obtained for 136 patients. The study was approved by the CHUSJ Ethics Committee for Health (References 259 and 260/11), in agreement with the Helsinki declaration. Informed consent was obtained from all the participants.

**Table 1:** Patients' clinicopathological information.

**Characteristics**	**No. of patients (%)**
Age, median (IQR)	70.5 (62.0–79.0)
Gender, M/F	83 (55.3)/67 (44.7)
**ANATOMIC TUMOR REGION**	
Cecum	11 (7.3)
Ascending colon	25 (16.7)
Transverse colon	21 (14.0)
Descending colon	11 (7.3)
Sigmoid	53 (35.3)
Rectum	29 (19.3)
**PATHOLOGICAL STAGE, TNM**	
Tumor	
T1	9 (6.0)
T2	25 (16.7)
T3	93 (62.0)
T4	23 (15.3)
Nodes	
N0	85 (56.7)
N+	65 (43.3)
Metastasis	
M0	121 (80.7)
M+	29 (19.3)
**CLINICAL STAGE**	
I	26 (17.4)
II	51 (34.0)
III	44 (29.3)
IV	29 (19.3)
**LYMPHOCYTIC INFILTRATION**	
Absent/mild	92 (61.3)
Moderate/strong	58 (38.7)
**ADJUVANT RADIOTHERAPY**	
No	135 (90)
Yes	14 (9.3)
Unknown	1 (0.7)
**ADJUVANT CHEMOTHERAPY**	
No	81 (54)
Yes	69 (46)
**RELAPSE**	
No	132 (88.0)
Yes	17 (11.3)
Missing	1 (0.7)
**SURVIVAL**	
Alive	76 (50.7)
Death	60 (40.0)
Unknown	14 (9.3)
**CAUSE OF DEATH**	
Cancer-related	29 (19.3)
Other causes	27 (18)
Missing	4 (2.7)

*IQR, interquartile range; M, male; F, female; No, number*.

### Immunohistochemical Staining

Specimens were fixed in formalin and embedded in paraffin in accordance with the routine protocol implemented at the Pathology Department from CHUSJ. Sequential 5 μm sections, from the most representative tumor region and selected by a Pathologist, were stained with antibodies against CD68 (Dako, PG-M1), CD80 (R&D, MAB140), and CD163 (Novocastra, MRQ-26). Briefly, tissues were deparaffinized, hydrated and endogenous peroxidase activity was blocked with 3% methanol in hydrogen peroxide for 10 min. Following antigen retrieval in a water bath at 98°C with Tris EDTA, pH9 (CD68, 20 min) or citrate buffer, pH6 (CD80, 20 min; CD163, 40 min), primary antibodies were incubated as follows: CD80 overnight (1:50) at 4°C, CD68 30 min (1:100) and CD163 30 min (1:100), both at room temperature. After washing, labeled polymer secondary antibody (Envision Detection System, Dako) was added to slides and peroxidase activity was detected using diaminobenzidine (DAB) –tetrahydrochloride liquid plus substrate Chromogen System (Dako). The reaction was stopped with distilled water and sections were counterstained with haematoxylin and mounted in Richard-Allan Scientific Mounting Medium (ThermoFisher).

### Macrophage Quantification

Following immunohistochemistry, the slides were digitalized using a NanoZoomer 2.0HT Hamamatsu camera (Meyer Instruments). For each marker, ten random areas of the ANM, IT, and IF were photographed (20x magnification). Using FIJI/IMAGEJ software, the immunoreactive area (IRA) for each cell surface marker and each region was calculated on the basis of red, green and blue segmentation, and represented as a percentage of the immunoreactive area (IRA%). Afterwards, the mean of the 10 distinct microscopic fields was calculated for each marker in each region. Importantly, the images of the three markers were acquired in the same area from consecutive sections.

### Statistical Analysis

Statistical analyses were conducted in STATA version 12.0 (StataCorp, College Station, Texas) or GraphPad Prism Software v5 (GraphPad-trial version). Departure from normality was determined using the Shapiro-Wilk test. Descriptive statistics included count and frequencies for categorical variables and median with interquartile range for continuous variables. Comparison of macrophage populations between and within locations in the tumor region was performed using Friedman's test followed by inter-group comparisons with Wilcoxon test. Comparisons between left and right colon side were performed with Mann-Whitney *U*-test while Kruskal-Wallis with Dunns multiple comparisons correction was applied in the analysis according to stage and primary tumor invasiveness. Kaplan-Meier plots with survival curves were compared with Log-rank test. The strength of associations between continuous variables was tested using Spearman's rank correlation. Association between macrophage populations and location with relapse followed a multistep statistical procedure: first, empirical analyses with unconditional logistic regression adjusting for age and gender, were carried out to uncover the relevant independent variables to be included in subsequent multivariate models (*p* for retention > 0.05); then, multivariate logistic regression was conducted to assess the independent strength of association of macrophage's characteristics in predicting risk for CRC progression. Lastly, in order to confirm the strength of association of the results emerging from multivariate analysis, bootstrapping analysis was performed using Monte Carlo simulations (*n* = 1,000).

## Results

### CD68^+^ and CD163^+^ Cells Are Predominantly Found Within the Tumor Invasive Front Whereas CD80^+^ Cells Are Mainly Located in the Tumor Adjacent Normal Mucosa

**Figure 1:**
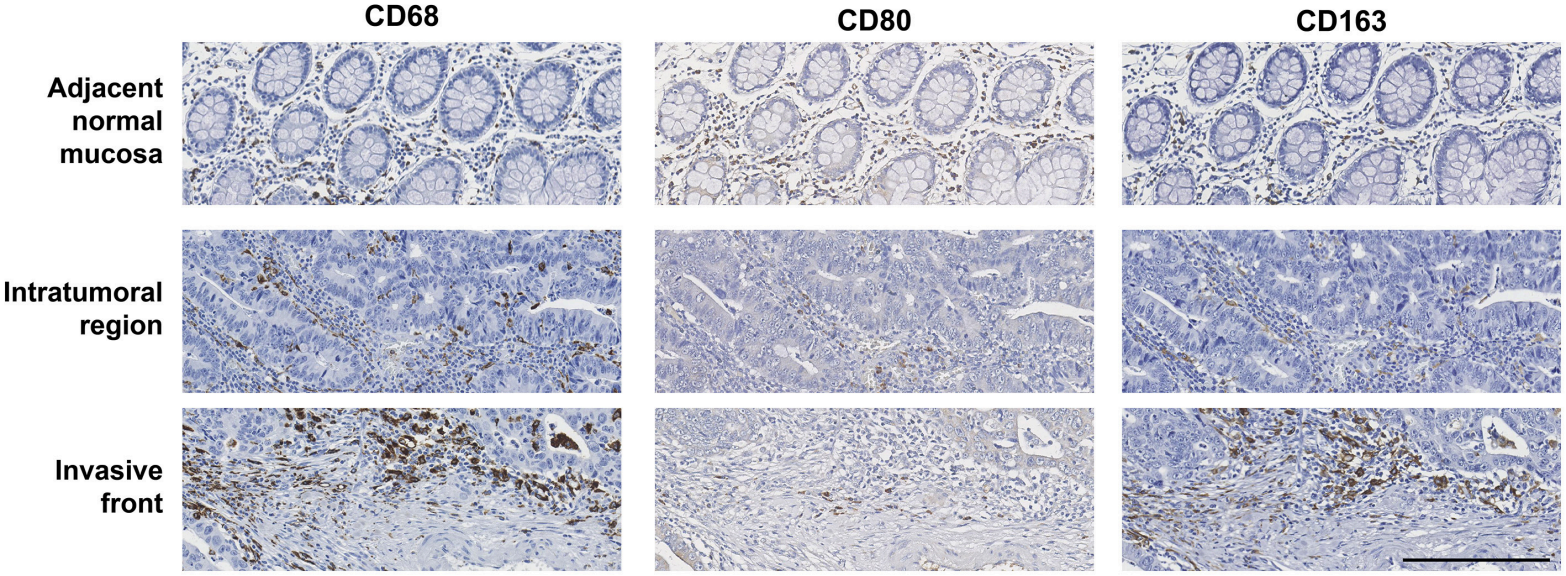
Immunostaining of CD68, CD80, and CD163 in the tumor adjacent normal mucosa, intratumoral region and invasive front of a representative colorectal cancer case, in consecutive paraffin-embedded sections. Specifically, it belongs to a stage IIa colorectal tumor in the ascending colon. Scale bar = 200 μm.

**Figure 2:**
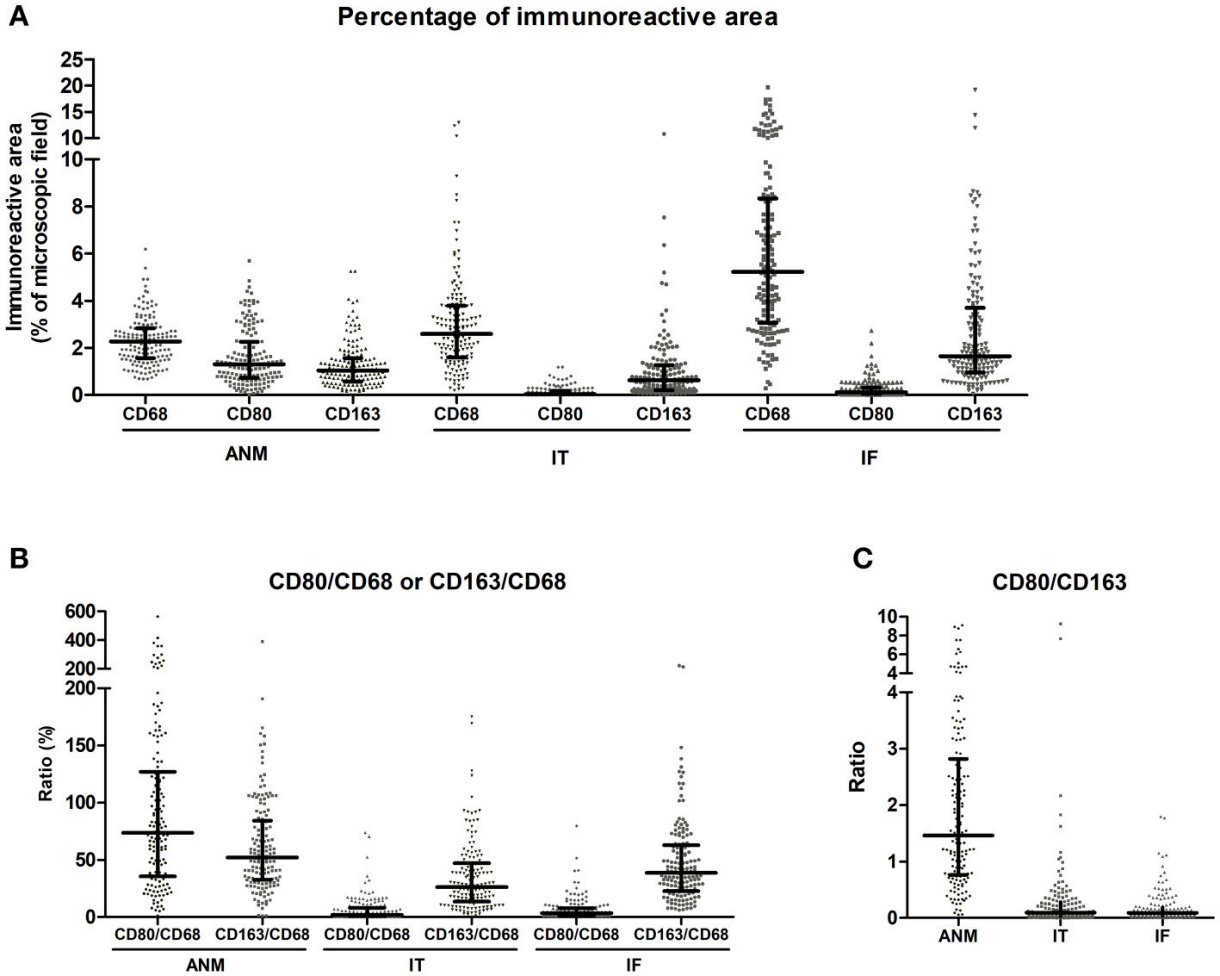
Quantifications of CD68, CD80 and CD163 in the 150 colorectal cancer cases. **(A)** Percentage of immunoreactive area (IRA%) of CD68, CD80, and CD163 in the tumor adjacent normal mucosa (ANM), intratumoral region (IT) and invasive front (IF). **(B)** Percentage of CD80/CD68 and CD163/CD68 ratios in the ANM, IT, and IF calculated from the IRA%. **(C)** CD80/CD163 ratio in the ANM, IT and IF calculated from the IRA%. Each dot represents one patient, calculated by averaging the quantification of 10 areas. Median and inter-quartile range are also included.

Given the difficulty in accurately assessing macrophage number using the classical approach of counting cells under the microscope, macrophage populations were evaluated by digitally quantifying the percentage of IRA%, similarly to what was carried out by other groups (Supplementary Figure 1) (27, 28). Three markers were used to characterize macrophages: CD68, a macrophage lineage marker broadly used to identify these immune cells (16, 18, 20), CD80, a co-stimulatory molecule expressed by pro-inflammatory macrophages (29), and CD163, a scavenger receptor associated with anti-inflammatory macrophages (30). Quantifications were performed in three regions: the ANM, the IT and the IF (Figure 1). Macrophages are mainly located at the IF of colorectal tumors comparing with the IT (5.23 vs. 2.59%) (18, 31), and the ANM (2.27%) (Figure 2A and Table 2). CD163^+^ cells are also predominantly found at the IF (1.65%), whereas the ANM exhibits a higher density of these anti-inflammatory cells than the IT region (1.04 vs. 0.63%) (Figure 2A and Table 2). Notably, CD80 is almost exclusively located in the ANM (1.31%). In the tumor regions, CD80 staining is very low and, similarly to the other markers evaluated, its expression is higher in the IF than in the IT (0.12 vs. 0.04%) (Figure 2A and Table 2). In the three regions analyzed, Spearman's rank correlation test revealed a moderate association between CD68 and CD163 staining (r_s_ >0.5), suggesting that tumors with higher levels of CD68 also present higher infiltration of CD163^+^ cells (Supplementary Table 1).

**Table 2:** Comparisons of percentage of immunoreactive area (IRA%) for CD68, CD80 and CD163, and CD80/CD68, CD163/CD68, and CD80/CD163 ratios in the adjacent normal mucosa, intratumoral region and invasive front.

	**Adjacent normal mucosa**	**Intratumoral region**	**Invasive front**	*p* value^*^
CD68 (IRA %)	2.27 (1.56–2.83)	2.59 (1.60–3.79)	5.23 (3.05–8.34)	*p* < 0.0001^a^
CD80 (IRA %)	1.31 (0.73–2.26)	0.04 (0.01–0.17)	0.12 (0.04–0.31)	*p* < 0.0001^b^
CD163 (IRA %)	1.04 (0.57–1.57)	0.63 (0.20–1.26)	1.65 (0.96–3.70)	*p* < 0.0001^c^
CD80/68 ratio (%)	73.75 (35.64–127.05)	2.06 (0.70–8.22)	3.45 (1.12–7.91)	*p* < 0.0001^d^
CD163/68 ratio (%)	51.98 (32.50–84.32)	26.16 (13.47–47.17)	38.69 (22.72–62.87)	*p* < 0.0001^e^
CD80/163 ratio	1.47 (0.76–2.82)	0.10 (0.03–0.28)	0.09 (0.04–0.19)	*p* < 0.0001^f^

*Data presented as median and inter–quartile range*.

* *Friedman's test. Group comparisons using the Wilcoxon test*.

a *ANM vs. IT (p = 4.70 × 10^−4^), ANM vs. IF (p = 3.65 × 10^−22^), IT vs. IF (p = 4.80 × 10^−19^)*.

b *ANM vs. IT (p = 8.11 × 10^−26^), ANM vs. IF (p = 2.36 × 10^−25^), IT vs. IF (p = 2.22 × 10^−9^)*.

c *ANM vs. IT (p = 5.36 × 10^−5^), ANM vs. IF (p = 1.55 × 10^−11^), IT vs. IF (p = 5.21 × 10^−21^)*.

d *ANM vs. IT (p = 2.30 × 10^−26^), ANM vs. IF (p = 3.05 × 10^−26^), IT vs. IF (p = 0.089)*.

e *ANM vs. IT (p = 1.97 × 10^−13^), ANM vs. IF (p = 1.46 × 10^−5^), IT vs. IF (p = 7.95 × 10^−9^)*.

f *ANM vs. IT (p = 2.45 × 10^−24^), ANM vs. IF (p = 2.76 × 10^−26^), IT vs. IF (p = 0.155)*.

*IRA, immunoreactive area*.

Since the quantifications for each marker were performed in consecutive sections of the same area, the percentage of pro-inflammatory and anti-inflammatory cells among the overall macrophage population was assessed calculating the ratio between CD80 and CD68 or CD163 and CD68 expression (Figure 2B and Table 2). Interestingly, at the ANM, CD80 staining represented almost 75% of the total CD68 staining. Of note, some of the cases studied had a higher CD80 IRA% compared with CD68, suggesting that CD80 is not exclusively expressed by macrophages. Within the IT and IF, the percentage of cells expressing CD80 relatively to CD68 decreased to ~2 and 3.45%, respectively. As for CD163, its expression represents about 50% of the total CD68 staining in ANM. Despite the increase of CD163^+^ cells at the IF, their percentage relatively to CD68 expression is still lower than what was observed in ANM (38.7%). Taken together, these observations demonstrate the presence of a significant number of macrophages at the IF and IT regions that do not express CD80 or CD163.

The ratio CD80/CD163 was also calculated to evaluate the proportion between pro- and anti-inflammatory macrophages (Figure 2C and Table 2). In the ANM, CD80 expression is 1.5 times higher compared to CD163. Conversely, both in IT and IF, CD163 expression is 10 times higher than CD80. Spearman's test revealed a positive association regarding CD80/CD163 ratio between IT and IF (*r*_s_ = 0.57) (Supplementary Table 1), suggesting that specimens with lower CD80/CD163 ratio at the IT region, are also the ones with a lower CD80/CD163 ratio at the IF.

### Adjacent Normal Mucosa and Tumors in the Right-Sided Colon Exhibit Higher Macrophage Infiltration

Given the known differences between the right and left-sided colon, not only in terms of anatomy and genetic alterations but also considering the microbiota present (32), macrophage populations in both locations were compared (Supplementary Table 2). Interestingly, CD68, CD80, and CD163 infiltration was higher in the ANM of tumors in the right than in the left-sided colon. Increased infiltration was also observed for CD68 and CD163 in the IT. Nevertheless, at the IF, the previously described differences between left and right-sided colon are lost for the three macrophage markers analyzed.

### Stage II Tumors Have Higher Infiltration of CD68^+^ and CD163^+^ Cells Whereas CD80^+^ Cells Are More Abundant in T1 Tumors

**Figure 3:**
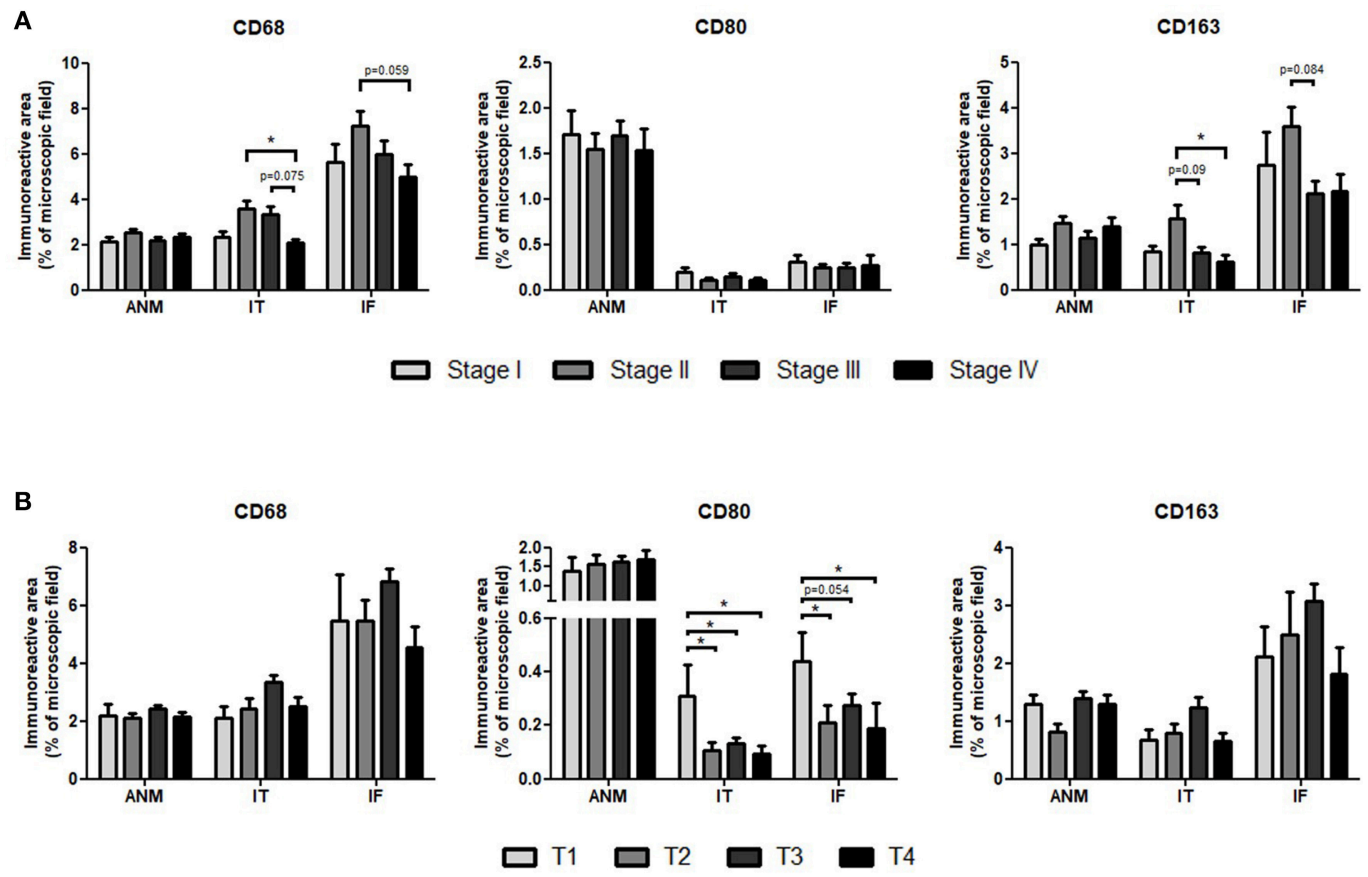
Percentage of Immunoreactive area of CD68, CD80, and CD163 in the adjacent normal mucosa (ANM), intratumoral region (IT) and invasive front (IF) according to **(A)** tumor stage or **(B)** primary tumor invasive depth. Results are presented as mean and standard error of the mean (SEM). Stage I = 26 patients; Stage II = 51 patients; Stage III = 44 patients; Stage IV = 29 patients. T1 = 9 patients; T2 = 25 patients; T3 = 93 patients; T4 = 23 patients. ^*^p < 0.05, Kruskal-Wallis with Dunns multiple comparisons.

Macrophage scores were then assessed according to tumor stage (Figure 3A). For the three markers analyzed, there were no differences in the ANM among the distinct CRC stages. Conversely, CD68^+^ and CD163^+^ macrophages were significantly more abundant at both IF and IT regions of stage II comparing with stage IV tumors. No differences were observed for CD80.

In a more profound analysis, macrophage populations were separately analyzed based on the primary tumor depth of invasion (Figure 3B). Interestingly, CD80^+^ cells were more frequent in the IT and IF of the less invasive T1 tumors. This was not observed in CD68^+^ or in CD163^+^ cells, which appear to predominantly infiltrate T3 tumors, although no statistical significant differences were detected.

### Higher CD68 Expression in Stage III Colorectal Tumors Is Associated With Decreased Overall Survival

**Figure 4:**
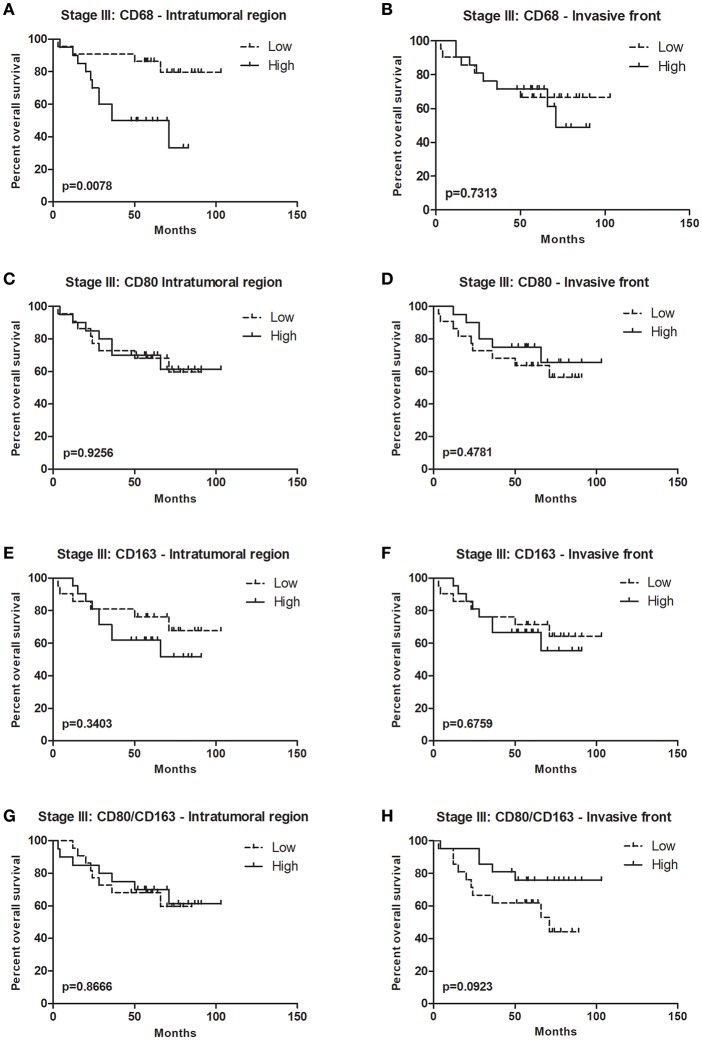
Overall survival curves for stage III colorectal cancer patients. Forty-four stage III CRC patients were divided into two groups, low, and high, according to the median of immunoreactive area percentage for each marker: **(A)** CD68, **(C)** CD80, **(E)** CD163, and **(G)** CD80/CD163 ratio in the intratumoral region, and **(B)** CD68 **(D)** CD80, **(F)** CD163, and **(H)** CD80/CD163 ratio in the invasive front. Kaplan-Meier plots and curves were compared through Log-rank test.

In CRC, the data regarding macrophage infiltration and patient survival is contradictory (17, 18). In order to perform this analysis, the IRA% for each marker was stratified into two categories according to the median, as low and high-expressing. When all patients were included in survival analysis, no differences were observed regardless of the marker or region analyzed (data not shown). Moreover, analyses conducted in colon cancer patients, excluding rectum malignancy, also yielded no relationship of markers and survival (data not shown). Given that our retrospective cohort includes all tumor stages, with different prognosis, the association between macrophages and survival was evaluated considering stages I + II, stage III, and stage IV separately. Specifically in stage III tumors, higher infiltration of CD68^+^ cells in the IT was associated with decreased overall survival (Figure 4A). This was no longer observed in the IF (Figure 4B), nor regarding CD80 or CD163 expression (Figures 4C–F). The association between patients overall survival and the CD80/CD163 ratio was also assessed. In stage III tumors, although not statistically significant, there seems to be an association between higher CD80/CD163 ratio in the IF and improved overall survival (Figure 4H). This result suggests that, in stage III, a higher proportion between pro and anti-inflammatory cells, may represent a survival advantage. It would be interesting to perform the same analysis in a bigger cohort to validate these results.

### Lower CD80 Infiltration Is Associated With Increased Relapse

Local recurrence is a frequent concern in CRC treatment (33) and efforts are being made to discover factors that might help predict such risk (34). Among the 150 cases of our series, 17 experienced relapse. No differences were detected in the percentage of CD68^+^ or CD163^+^ macrophage infiltration between patients with vs. without relapse, in the three regions analyzed. Conversely, specimens from patients without relapse, presented a significantly higher CD80 IRA% in both the IT (*p* = 0.016) and in the IF (*p* = 1.16 × 10^−7^). Univariate logistic regression revealed an association between higher CD80 staining at the IF and a decreased risk for relapse (Table 3). This finding was further confirmed on multivariate logistic regression that included only variables with significant risk and validated by bootstrap analysis (Supplementary Table 3). Overall, these results support a protective role of CD80^+^ cells at the IF of colorectal tumors for relapse.

**Table 3:** Empirical univariate analysis of independent variables, clinicopathological and macrophage markers, in association with risk for disease relapse with adjustment for age and gender.

	**Risk for disease relapse**	
**Variables**	**OR (95 CI)**	***p* value**
Tumor anatomic region	**1.60 (1.04–2.40)**	**0.032**
Clinical stage	0.68 (0.30–1.90)	0.461
Radiotherapy	**18.2 (5.30–61.90)**	**<0.0001**
Chemotherapy	3.00 (0.94–9.50)	0.062
ANM^*^		
CD68	0.92 (0.55–1.55)	0.757
CD80	0.85 (0.53–1.37)	0.502
CD163	0.91 (0.52–1.61)	0.757
IT^*^		
CD68	1.09 (0.87–1.36)	0.453
CD80	0.02 (0.00–4.40)	0.153
CD163	0.74 (0.40–1.38)	0.346
IF^*^		
CD68	0.93 (0.81–1.08)	0.365
CD80	**0.001 (0.00–0.48)**	**0.030**
CD163	0.82 (0.61–1.13)	0.191

OR, odds ratio; 95CI, 95% confidence interval; ANM, adjacent normal mucosa; IT, intratumoral region; IF, invasive front;

* *analyzed as continuous variables. Statistical significant associations are marked in bold*.

Surprisingly, radiotherapy revealed a significant association with increased risk of relapse in multivariate analyses, further confirmed through bootstrapping. This may be related, not to the therapy itself, but to the specific characteristics of the colorectal tumors candidate for this therapeutic approach.

## Discussion

Innate immune cells present at the tumor microenvironment may participate in several stages of cancer progression (28, 35, 36). In particular, TAMs play an important role in tumorigenesis (4) and, although controversy, high levels of macrophage infiltration have been associated with poor prognosis and reduced therapy response, in distinct types of tumors.

In CRC, increased infiltration of lymphocytic cells correlates with improved clinical outcome. Higher infiltration of T cells (CD3^+^), cytotoxic T cells (CD8^+^), and memory T cells (CD45RO^+^) has been associated with longer disease-free and/or overall survival (37). Moreover, the Immunoscore, based on the quantification of lymphocyte populations (CD3/CD8, CD3/CD45RO, or CD8/CD45RO), demonstrated higher robustness and prognostic value than the classical UICC's TNM classification for stages I-III. In agreement, this immune-based classification is currently being introduced into clinical settings (38–40). Despite being the most represented immune population in solid tumors, macrophages are not included in this classification, likely due to contradictory results in studies addressing their clinicopathologic significance in CRC.

In this study, macrophage profiling was assessed by quantitative evaluation of a macrophage lineage marker (CD68), a co-stimulatory receptor expressed by pro-inflammatory macrophages (CD80) and a scavenger receptor characteristic of their anti-inflammatory counterparts (CD163). The latter has been previously described in the literature, including in studies performed in CRC (23, 41–43). However, the identification of an ideal pro-inflammatory macrophage marker has been more challenging. Although several reports used NOS2 (22, 23, 44), it is becoming more evident that this is a specific marker of mice but not of human pro-inflammatory macrophages (25, 26). Our preliminary *in vitro* analysis revealed that CD80, referred in the literature as specifically expressed by M1 macrophages (45), and previously used to identify this specific subpopulation in tumors (46), is a suitable alternative (Supplementary Figure 2). Nevertheless, none of these markers is completely specific and it is possible that other immune cell populations, namely monocytes, dendritic cells, or activated B cells, are also recognized.

This study demonstrates that macrophage subpopulations are not uniformly distributed along the tumor, with distinct preferences for ANM, IT and IT regions. Our results showed increase CD68 staining in tumors compared to ANM, supporting the idea that these cells migrate towards the tumor site by chemotactic signals (47, 48). Although CD80 was highly expressed by macrophages at the tumor ANM (~74%), the majority of macrophages in intratumor regions lack the expression of this pro-inflammatory marker. This observation does not corroborate other studies performed in CRC using NOS2 as a marker, in which ~60% of the overall tumor macrophages were considered pro-inflammatory (23), but again we argue that this might not be an ideal pro-inflammatory marker of human macrophages. In accordance with the literature, we confirmed that the IF of colorectal tumors was densely infiltrated by macrophages and that, of these, <40% were CD163^+^ cells (23). We further demonstrated that only 3.5% of the IF macrophages stained positively for CD80. These results evidence an alteration of the macrophage inflammatory profile from the ANM to the neoplastic regions, with a major reduction of the CD80 expression, not counterbalanced by an increase of the CD163^+^ cells. Moreover, it also indicates that more than half of the TAMs are not expressing any of the polarization markers selected. Additionally, we cannot exclude that some macrophages might be expressing both M1 and M2 markers (22). Given the broad spectrum of M2-macrophages (5), these results highlight the heterogeneity in TAMs within CRC. A potential marker to include in future analysis is CD206. Work by Norton and colleagues describing TAMs subsets in CRC through flow cytometry showed distinct populations expressing CD163 and/or CD206 (49). More recently, Feng et al. also evidenced that, within stage II CRC patients which underwent radical resection, CD206/CD68 ratio can identify those with high risk of recurrence and poor prognosis and might benefit from adjuvant chemotherapy (50). In other models, namely esophageal squamous cell carcinoma, it was shown that there is a subpopulation of TAMs that does not express CD163 but is positive for CD204 (51). In fact, macrophage plasticity and ability to shift between polarization statuses represents a true challenge for their characterization. In the future, it will be of upmost importance to characterize macrophages not recognized by CD80 or CD163 antibodies, by isolating CD68^+^CD80^−^CD163^−^ cells from formalin-fixed paraffin-embedded tissues and performing extensive gene expression analysis (52), to identify other subpopulations with putative relevant prognostic value or as novel targets for therapeutic modulation. As previously described by our group, one of the key players that might be determining these differential macrophage polarization within colorectal cancer is the extracellular matrix. By using decellularized human CRC and non-neoplastic mucosa, we demonstrated that, contrarily to what happens in normal tissues, tumor-ECM polarizes macrophages toward an anti-inflammatory, pro-invasive phenotype (53).

Interestingly, when macrophage populations were evaluated according to tumor stage, CD68 and CD163 expressing cells were more abundant in stage II tumors in comparison to stage IV, in agreement to findings from Sickert et al. (47). Conversely, Bailey et al. reported a higher macrophage infiltration in stages III and IV, but this study included a small series of patients (54). Concerning CD80 staining, in our cohort, no differences were observed among tumor stages. It is generally hypothesized that during the initial steps of tumor development, macrophages recruited to the tumor site acquire pro-inflammatory and anti-tumor activities. Then, as a result of increased IL-10 and TGF-β levels, their polarization shifts toward a pro-tumor anti-inflammatory phenotype (45). For this reason, macrophage populations were separately analyzed according to the primary tumor invasiveness depth. Noteworthy, we observed that specifically CD80^+^ cells were predominant in T1 tumors, supporting, to some extent, the previously mentioned hypothesis.

Significant differences between ascending and descending colon might be partially explained by embryological origin: while ascending colon derives from the midgut, the descending colon is originated in the hindgut. Work by Glebov et al. reporting gene expression analysis of the ascending and descending normal colon mucosa from the same subject, revealed clear differences in the expression of genes involved in the control of many cellular functions, namely cell proliferation, adhesion, death, and signal transduction. Moreover, by including fetal samples in their study, they concluded that, although significant differences are indeed already established in the embryonic colon, additional alterations in gene expression arise in postnatal development (55). The gut microbiome has also been a subject of thorough investigation and it is now known that the amount and type of bacteria in the ascending and descending colon are distinct (56). These differences might be reflected in our findings, since CD68^+^, CD163^+^, and CD80^+^ macrophages were more prevalent in the tumor ANM on the ascending side of the colon. The same was observed in the IT region for both CD68 and CD163. Besides macrophages, other immune cells, namely some T lymphocytes subpopulations have also been reported as predominant in ascending colorectal tumors (57). Moreover, it is described that right-sided tumors have an higher mutational load which may be involved in the increased recruitment of immune cells (58). Interestingly, the differences disappeared at the tumor IF, suggesting that, in this specific region, the tumor can modulate the immune response regardless of the initial environment. This might be related with the different chemokines tumor cells release, which are known to have an impact in immune cell recruitment and differentiation (59). Our unexpected results suggesting that radiotherapy associates with increased risk of relapse are probably not related to the direct effect of ionizing radiation but, more likely, to the endogenous molecular characteristics of the tumors recommended for radiotherapy treatment. These results should be exploited in future studies.

In terms of prognosis, our results indicate that, within stage III tumors, higher CD68 infiltration in the IT is associated with decreased overall survival, contrarily to what was reported by Malesci et al. Using stage III CRC patients, they reported an association between high CD68 infiltration and increased overall survival, but only in patients undergoing 5-FU treatment (28). Our results further revealed an association between higher CD80/CD163 ratio at the tumor IF and improved survival, similarly to what was reported in ovarian cancer (60). Recent work by Yank and colleagues describes an association between higher CD163^+^/CD68^+^ ratio at the IF of colorectal tumors and poor prognosis, which is not in accordance with our data. It would be important to clarify if the quantifications of CD68 and CD163 were performed in exactly the same tumor regions (61). This work strengthens the need to establish the inflammatory profile of existing macrophage populations and to perceive their distribution at the tumor microenvironment for an accurate prognostic prediction, and possible, therapeutic intervention. Different strategies targeting macrophages are currently under development (62), namely inhibition of monocyte recruitment (63), or of macrophage activation (64). More recently, the possibility of reprogramming M2 macrophages toward the M1-type has also been considered (65). In this sense, recent work by our group described the immunomodulatory capacity of polyelectrolyte multilayers containing IFN-γ and of nanoparticles composed of polyglutamic acid, specifically in reverting the pro-invasive capacity of IL-10-stimulated macrophages (66, 67). Accordingly, it is plausible to speculate that CRC patients might benefit from a therapeutic strategy aiming at reprogramming TAMs profile, which would result in an increase of M1 macrophages with a concomitant decrease of M2 subpopulations. The potential of this approach is further strengthen by the observation that lower infiltration of CD80^+^ cells strongly associated with increased risk of relapse. In hepatocellular carcinoma, an increase in M1 macrophages associated with increased time until recurrence (68), and a reduced CD163/CD68 ratio was correlated with a worse outcome (68), which corroborates, at least partially, our results. Specifically in CRC, a gene-expression based study published last year shows that tumors lacking M1 macrophages are associated with poor prognosis (69). Recent work revealed that CRC cells co-cultured with M1 macrophages exhibited increased cell death. Conversely, in the presence of naïve, unstimulated macrophages, cell death remained unchanged or even decreased, depending on the cell line (28). Nevertheless, since this the is first report describing the protective role of CD80^+^ cells in preventing CRC relapse, further studies should be performed to validate the current findings. Moreover, given the described reduced risk of relapse in colorectal tumors with higher immunoscore, it would be important to explore possible associations between the infiltration of CD80^+^ cells and cytotoxic or memory T cells.

Altogether, this work contributed to increase the knowledge regarding macrophage profile in CRC and further reinforced the complexity of macrophage polarization in tumors. Macrophage intrinsic plasticity and the capacity to adopt intermediate profiles between the two extreme populations, the M1 and the M2, require the use of multiple markers and a combination of strategies to accurately dissect the overall macrophage phenotype in tumors. The association of lower CD68 infiltration and higher CD80/CD163 ratio with increased overall survival within stage III CRC supports the need for further validations and reinforces the relevance of including such markers in the already established Immunoscore. Furthermore, the possible protective role of CD80^+^ cells in preventing relapse might also open new perspectives in the immunotherapy field. Results presented here further support the need to foster research focusing on the development of novel therapeutic strategies to reprogram macrophages toward the pro-inflammatory and tumoricidal phenotype (70).

## Data Availability

The datasets generated for this study are available on request to the corresponding author.

## Author Contributions

MP, FC, and MO conceived and designed the study. The experimental procedures, data analysis, and original draft writing were performed by MP. ER collected human colorectal samples, prepared histological sections, and contributed to pathological analysis. CD and RR assisted all statistical analysis and data interpretation. AM, MB, and JM contributed to data interpretation and discussion. FC supervised and monitored pathological data interpretation. MO supervised data analysis and discussion and obtained financial support. All authors discussed the results, contributed to the writing of the manuscript, and revised the final version.

### Conflict of Interest Statement

The authors declare that the research was conducted in the absence of any commercial or financial relationships that could be construed as a potential conflict of interest.
