# Supplementary material for: The Two Faces of Tumor-Associated Macrophages and Their Clinical Significance in Colorectal Cancer
Source: Front Immunol. 2019 Aug 20;10:1875. doi: 10.3389/fimmu.2019.01875 (PMC6710360; doi:10.3389/fimmu.2019.01875)
Supplement: Supplementary file 1 [file Data_Sheet_1.pdf]

Supplementary data

Supplementary materials and methods

Monocyte isolation and polarization

Human monocytes were isolated from Buffy coats from healthy blood donors provided by Hospital São João. Briefly, buffy coats were centrifuged at 1200g for 20 minutes at room temperature. The whitish layer containing peripheral blood mononuclear cells (PBMCs) was collected and incubated, during 20 minutes under continuous rotation, with the RosetteSep Human Monocyte Enrichment Cocktail (StemCell Technologies), following manufacturer's instructions. This mixture was diluted (1:1) in PBS+2% FBS, carefully added over Histopaque-1077 (Sigma), and centrifuged as previously. The intermediate layer, enriched in human monocytes, was collected and washed three times in PBS. Isolated cells were then ressuspended in RPMI1640 (Invitrogen) supplemented with 10% FBS (Lonza), 100U/ml penicillin and 100µg/ml streptomycin (Invitrogen). Cells were seed in coverslips, allowed differentiate during 10 days and were then treated with 10ng/ml of LPS (Sigma) or IL-10 (Immunotools), for three additional days, to induce their polarization into M1 or M2 macrophages, respectively. After treatment, cells were fixed with methanol for 10 minutes at 4˚C.

Immunocytochemistry

After washing three times with PBS, cells were blocked for 30 minutes with 5% bovine serum albumin (BSA). The same primary antibodies used in the IHC, CD80 or CD163, were incubated for one hour. After washing with PBS, coverslips were incubated for an additional hour with goat anti-mouse AlexaFluor-594-conjugated secondary

antibody. Samples were finally washed with PBS and coverslips were mounted on Vectashield with DAPI. Cells were visualized with a Zeiss Axiovert 200M fluorescence microscope.

Supplementary figures

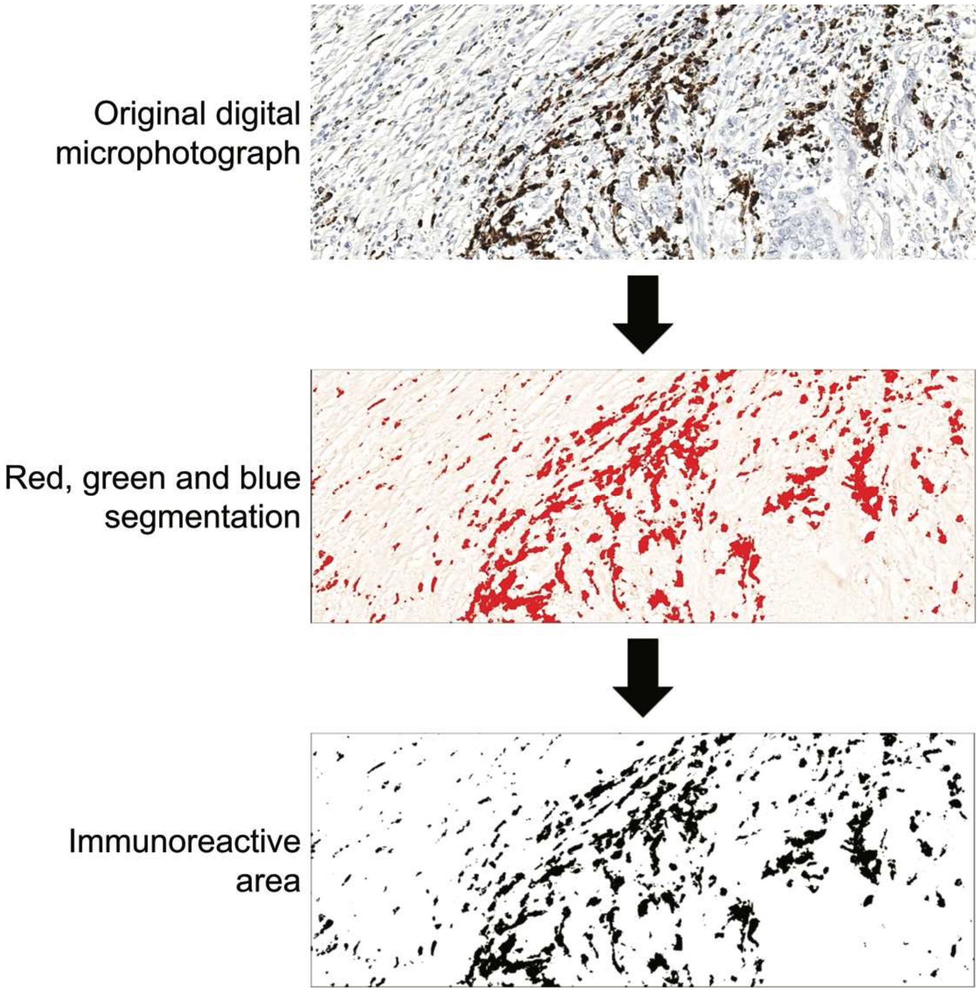

Supplementary figure 1. Scheme representing the steps followed for the quantification of the percentage of immunoreactive area. Example of computer assisted quantification of the immunoreactive area based on red, green and blue (RGB) segmentation from an original photograph. The immunoreactive area percentage is automatically quantified in relation to the total image area.

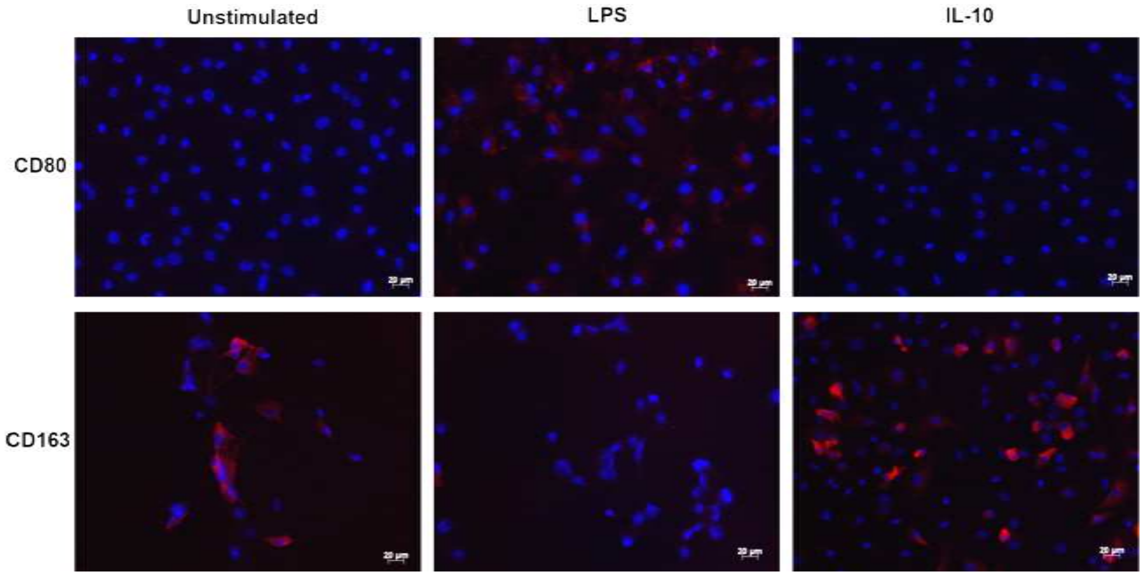

Supplementary figure 2. LPS-stimulated macrophages express CD80 while IL-10 stimulated ones express CD163. Human monocyte-derived macrophages were stimulated with LPS or IL-10, in order to induce M1 or M2 polarization, and stained with CD80 or CD163 antibodies. Unstimulated cells were used as control. Scale bar represents 20μm.

Supplementary tables

Supplementary table 1. Spearman's rank correlation for the percentage of immunoreactive area for CD68, CD80 and CD163 in the adjacent normal mucosa (ANM), intratumoral region (IT) and invasive front (IF).

		Normal			Intratumoral region			Invasive front		
		**CD68**	**CD80**	**CD163**	**CD68**	**CD80**	**CD163**	**CD68**	**CD80**	**CD163**
**Normal**	**CD68**		0.14 p=0.081	**0.51** p<0.0001	0.35 p<0.0001	-0.11 p=0.184	0.21 p=0.01	0.25 p=0.002	0.00 p=0.963	0,23 p=0,004
	**CD80**			0.41 p<0.0001	0.1 p=0.231	0.18 p=0.026	0.20 p=0.013	-0.10 p=0.238	0.27 p=0.001	0.07 p=0.37
	**CD163**				0.21 p=0.009	0.09 p=0.255	0.41 p<0.0001	0.01 p=0.89	0.15 p=0.071	0.28 p=0,001
**Intratumoral region**	**CD68**					0.19 p=0.018	**0.55** p<0.0001	0.46 p<0.0001	0.15 p=0.076	0.40 p<0.0001
	**CD80**						0.39 p<0.0001	-0.07 p=0.367	0.48 p<0.0001	0.07 p=0.425
	**CD163**							0.27 p=0.001	0.27 p=0.001	**0.61** p<0.0001
**Invasive front**	**CD68**								0.10 p=0.211	**0.64** p<0.0001
	**CD80**									0.35 p<0.0001
	**CD163**									

Spearman Rank-order Coefficient (rs) are presented together with the p values. rs>0.5 are depicted in bold.

Supplementary table 2. Percentage of immunoreactive area of CD68, CD80 and CD163 in the adjacent normal mucosa, intratumoural region and invasive front in the right and left-sided colon.

Region	Marker	Right colon, N=52	Left colon, N=98	p value
**Adjacent normal mucosa**	**CD68**	2.56	2.20	0.022
	**CD80**	2.02	1.40	0.002
	**CD163**	1.71	1.04	0.0004
**Intratumoral region**	**CD68**	3.66	2.65	0.004
	**CD80**	0.15	0.12	0.466
	**CD163**	1.36	0.86	0.0705
**Invasive front**	**CD68**	5.82	6.37	0.392
	**CD80**	0.29	0.25	0.122
	**CD163**	3.08	2.54	0.158

Data are represented as mean of immunoreactive area percentage. p values were obtained by Mann-Whitney U test.

Supplementary table 3. Multivariate stepwise logistic regression (pr<0.05) including only variables with significant risk from univariate analysis (Table 3). In order to confirm the strength of association of emerging results from multivariate analysis, bootstrap analysis using Monte Carlo simulations (n=1000) was performed.

	**Multivariate**		**Bootstrap**	
	OR (95 CI)	p value	OR (95 CI)	p value
**Radiotherapy**	**17.77 (4.83-65.29)**	**<0.0001**	**19.93 (2.55-155.49)**	**0.004**
**IF_CD80***	**0.001 (0.00-0.92)**	**0.047**	**0.001 (0.00-0.45)**	**0.028**

OR, odds ratio; 95 CI, 95% confidence interval; IF, tumor invasive front; * analyzed as continuous variables.
